# Analysis of the association between areal socioeconomic deprivation levels and viral hepatitis B and C infections in Japanese municipalities

**DOI:** 10.1186/s12889-022-13089-w

**Published:** 2022-04-07

**Authors:** Tasuku Okui, Naoki Nakashima

**Affiliations:** Medical Information Center, Kyushu University Hospital, Fukuoka city, 812-8582 Maidashi3-1-1 Higashi-ku, Fukuoka, Japan

**Keywords:** Screening, Japan, Hepatitis, Viruses, Health Status Disparities

## Abstract

**Background:**

We investigated the association between municipal socioeconomic deprivation levels and the positivity of hepatitis B surface antigen (HBsAg) and the prevalence of hepatitis C virus (HCV) among individuals who have never participated in hepatitis screening using Japanese national screening data.

**Methods:**

The hepatitis virus screening data analyzed included the 5-year age group-specific number of participants aged 40 years or older, number of HBsAg-positive persons, and number of HCV carriers for each municipality from 2013 to 2017. Principal component analysis was used to derive a socioeconomic deprivation level using the socioeconomic characteristics of municipalities. Bayesian spatial Poisson regression analysis was conducted to investigate the association between the socioeconomic deprivation level and the results of screening. Data on 1,660 municipalities were used in the analysis.

**Results:**

The data of 4,233,819 participants in the HBV screening and 4,216,720 in the HCV screening were used in the analysis. A principal component interpreted as level of rurality (principal component 1) and another principal component interpreted as level of low socioeconomic status among individuals (principal component 2) were extracted as the major principal components. Their principal component scores were used as the deprivation levels of municipalities. Spatial regression analysis showed that the deprivation level derived from the sum of the scores of principal components 1 and 2 was significantly and positively associated with HBsAg positivity and HCV prevalence. In addition, the deprivation level derived only from the score of principal component 2 was also significantly and positively associated with the outcomes. Conversely, the deprivation level derived only from the score of principal component 1 was not associated with the outcomes. Moreover, population density was significantly and positively associated with HBsAg positivity and HCV prevalence.

**Conclusions:**

This study suggested that participation in hepatitis virus screening is important and meaningful, particularly for areas with a higher lower socioeconomic level in Japan.

**Supplementary Information:**

The online version contains supplementary material available at 10.1186/s12889-022-13089-w.

## Background

Hepatitis B virus (HBV) and hepatitis C virus (HCV) infections are major global public health issues, with an estimated a few hundred million infected people for each virus [1, 2]. HBV and HCV are major causes of liver diseases, such as cirrhosis and cancer [3]. The prevalence of HBV declined from the 1990s to the 2000s, possibly because of increased immunization [4]. However, there are regional differences in HBV and HCV infection worldwide [1, 4]. In Japan, administrative measures, such as HBV screening of all pregnant women and national hepatitis screening of the general population, have been implemented in recent decades for eliminating hepatitis virus infection [5]. As a result, the prevalence of HBV and HCV has decreased over Japanese birth cohorts [5]. Mortality related to HCV has also decreased in recent decades [6]. On the other hand, there are still a few million estimated carriers of HBV or HCV in Japan [5]. The early detection of undiagnosed patients and prevention of new infections are warranted.

Individual and regional socioeconomic status are related to HBV and HCV infection [7–9], and the prevalence of the viruses is particularly high in lower- and middle-income countries [1, 10]. A study investigating factors associated with the spatial distribution of HCV prevalence in the Netherlands, found that one-person households, non-Western immigrants, and divorced persons comprised the high-risk population [11]. In addition, a study in India found that a lower socioeconomic status and illiteracy were associated with HCV positivity [12]. Moreover, a study in Turkey found that self-employed persons, business owners, and public sector workers were less likely to be infected with HBV compared with labourers [13].

In Japan, in contrast, differences in the prevalence of HBV and HCV depending on individual or regional socioeconomic status have not been investigated, and it is meaningful to verify the association also in Japan. One way to determining the association between socioeconomic status and virus prevalence is statistically analyzing the association between the prevalence and regional socioeconomic status. In Japan, there are studies investigating regional differences in the prevalence of HBV and HCV [5, 14], and high-prevalence regions or prefectures have been shown. On the other hand, differences in prevalence by municipality across Japan have not been investigated. We could more accurately analyze regional socioeconomic positions and disease burdens by revealing municipal differences in disease prevalence or mortality, as in previous studies [15, 16]. Socioeconomic deprivation level is often calculated using multiple socioeconomic characteristics of regions in ecological studies [17, 18]. If a disparity in prevalence related to areal socioeconomic level exists in Japan, effective administrative measures in more deprived areas will help reduce HCV and HBV prevalence. For example, more encouragement of hepatitis screening or hepatic function test in those regions is meaningful and recommended in this case. In Japan, although data on HBV prevalence are not publicly available, data on positivity of hepatitis B surface antigen (HBsAg) are publicly available.

In this study, we investigated the association between areal socioeconomic deprivation levels and the positivity of HBsAg and the prevalence of HCV using national screening data from Japanese municipalities.

## Methods

### Data

We used hepatitis virus screening data from each municipality, collected based on the Health Promotion Act in Japan [19]. The screening results are gathered by the Ministry of Health, Labor, and Welfare and published in the Report on Regional Public Health Services and Health Promotion Services [19]. The target population for the screening was the general population aged 40 years or more. Individuals apply for the screening by themselves, and only individuals who had never participated in hepatitis virus screening could participate [20]. The HBsAg test is used for HBV screening. For HCV screening, the HCV antibody test is conducted first, and participants with a high titer are regarded as high-probability HCV carriers in the screening. Participants with middle or low titers undergo the qualitative HCV RNA test (HCV RNA), and individuals with positive results are also regarded as high-probability HCV carriers in the screening. The two types of high-probability HCV carriers were defined as HCV carriers in this study. Diagnostic criteria and implementation method of the screening for each municipality is established by the Ministry of Health, Labor, and Welfare [20]. Reference value of the high titer is 50 or more in Chemiluminescent enzyme immunoassay [CLEIA], and that of middle or low titers is 1–50 in the CLEIA [21].

Data on 5-year age group-specific number of participants, number of HBsAg positive persons, and number of HCV carriers for each municipality from 2013 to 2017 were used in the analysis. Classification of participants by municipalities is based on address of the participants. We aggregated the data from 2013 to 2017 for each municipality in the analysis because the numbers of HBsAg positive people and HCV carriers were relatively low. Data on municipalities whose number of participants, HBsAg positive people, or HCV carriers was completely or partially unknown were not used in the analysis.

As socioeconomic characteristics used for deriving socioeconomic deprivation levels, we used 7 municipal socioeconomic characteristics. Descriptions and the data sources of the characteristics, as well as other characteristics of municipalities used for regression analysis, are shown in Table 1. All the data except for the proportions of divorced persons and households living in rental housing were obtained from the general counter of government statistics in Japan [22]. The data of the proportions of divorced persons and households living in rental housing were obtained from the Census website [23]. All the data on socioeconomic characteristics in 2015 were used in the analysis except for the proportion of persons with lower educational level. Educational level was not investigated in the Census in 2015, and the data in 2010 were used in the analysis. In addition, map data of Japan were obtained from the digital national land information published by the Ministry of Land, Infrastructure, Transport, and Tourism [24].

**Table 1:** Socioeconomic characteristics used for deriving socioeconomic deprivation level

Variables	Description	Source
Socioeconomic characteristics		
Proportion of fatherless households	Proportion of fatherless households among total households (%)	The Census
Proportion of divorced persons	Proportion of divorced persons among persons aged 15 years old or more (%)	The Census
Proportion of persons with low educational level	Proportion of persons with elementary or junior high school graduates among persons aged 15 years old or more (%)	The Census and the Basic Resident Register
Proportion of labourers	Proportion of labourers in the labor force (%)	The Census
Proportion of unemployed persons	Proportion of unemployed persons in the labor force (%)	The Census
Taxable income per capita	–	The Survey on Taxation Status of Municipal Tax and the Basic Resident Register
Proportion of households living in rental housing	Proportion of households living in rental housing among total households (%)	The Census
Other characteristics		
Population density	Population per hectare	The Basic Resident Register and the Municipalities Area Statistics in Japan
Proportion of non-Japanese persons	Proportion of non-Japanese (%)	The Basic Resident Register
Proportion of elderly households	Proportion of elderly households among total households (%)	The Census
Proportion of single households	Proportion of single households among total households (%)	The Census
Proportion of self-employed persons	Proportion of self-employed persons among labor force population (%)	The Census
Number of hospitals per capita	Number of hospitals per 100,000 persons	The Survey of Medical Institutions and the Basic Resident Register
Number of medical clinics per capita	Number of medical clinics per 100,000 persons	The Survey of Medical Institutions and the Basic Resident Register

### Statistical analysis

An ecological study was conducted to investigate the association between the socioeconomic deprivation level and the number of HBsAg positive persons among municipalities. We calculated the age group-specific HBsAg positive rate (proportion of HBsAg-positive participants to the total number of participants) and HCV prevalence (proportion of HCV carriers to the total number of participants) for all of Japan. Then, we calculated the expected number of HBsAg-positive persons and HCV carriers for each age group and municipality using the age group-specific number of screening participants for each municipality and the national rates in Japan. We calculated the expected number of HBsAg-positive persons and HCV carriers for each municipality by summing the expected age-specific number. By using the actual and expected numbers of HBsAg positive persons and HCV carriers, we derived the standardized HBsAg positive ratio and standardized HCV prevalence ratio for each municipality, like the standardized mortality ratio. We used the empirical Bayes method for the calculation [25], and the R package DCluster (https://cran.r-project.org/web/packages/DCluster/DCluster.pdf) was used. The standardized HBsAg positive ratio and the standardized HCV prevalence ratio of municipalities were mapped.

For deriving the socioeconomic deprivation level, we conducted a principal component analysis using the socioeconomic characteristics [26]. All the socioeconomic variables were scaled before the principal component analysis. Then, we calculated the deprivation level by summing the principal component scores whose eigenvalues were above 1, as conducted in previous studies [27]. In addition, we calculated the deprivation level based on each of the principal component scores whose eigenvalues were above 1 and also used them in the analysis.

Although the standardized HBsAg positive ratio and the standardized HCV prevalence ratio are useful for comparing HBsAg positivity and HCV prevalence among municipalities, a relative risk cannot be calculated when using them in a regression analysis. Therefore, a Poisson regression model using the number of HBsAg-positive persons as the outcome variable and the socioeconomic deprivation level, as well as other characteristics, as the explanatory variables was conducted to calculate the relative risk between the socioeconomic deprivation levels. In addition, spatial autocorrelation often exists in spatial data. Therefore, we used a Bayesian spatial Poisson regression model called the Besag, York, and Mollié model by CARBayes [28], using the neighborhood matrix of municipalities. In the Bayesian spatial Poisson regression analysis, the expected number of HBsAg positive persons for each municipality was used as the offset variable for adjusting differences in population and age distribution among municipalities. The deprivation level as well, as the other characteristics of municipalities, were used as explanatory variables, and all the variables were standardized. The same analysis was conducted using the number of HCV carriers as the outcome. In addition, an analysis using each type of deprivation level derived from the principal component analysis was also conducted. All statistical analyses were conducted using R3.6.3 [29].

Institutional review board approval was not mandatory for this study because we used only publicly available data. All the works were carried out in compliance with relevant laws and guidelines. and with the ethical standards of the Declaration of Helsinki.

## Results

Data from 1,665 for HBV screening and data from 1,668 municipalities for HCV screening were available, and the data from 1,665 municipalities were available for both the results of HBV and HCV screening from 2013 to 2017. We used the data from 1,660 municipalities in the analysis after removing municipalities where number of residents was zero or extremely small because of an evacuation following the Great East Japan Earthquake. The total number of participants in the data was 4,233,819 for HBV and 4,216,720 for HCV screening.

**Fig. 1:**
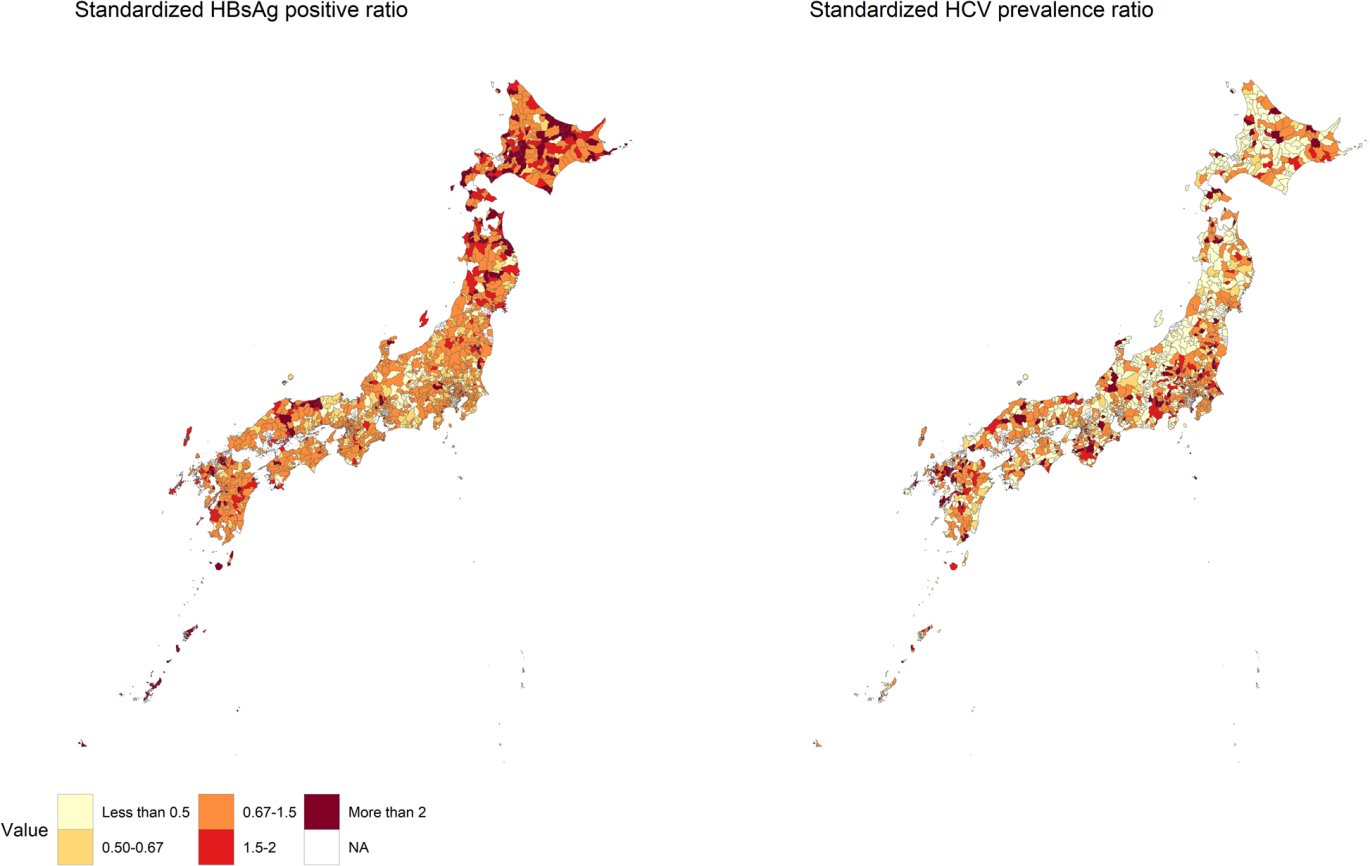
Geographic differences in the standardized HBsAg positive ratio and standardized HCV prevalence ratio in Japan. HBsAg, hepatitis B surface antigen; HCV, hepatitis C virus; NA, not available. The standardized HBsAg-positive ratio and the standardized HCV prevalence ratio are indexes taking into account of difference in age distribution of participants among regions like the standardized mortality ratio. If the standardized HBsAg-positive ratio in a region is high, it indicates that the ratio of HBsAg-positive participants to the total number of participants is high in the region. Similarly, if the standardized HCV prevalence ratio in a region is high, it indicates that the ratio of participants infected with HCV to the total number of participants is high in the region

Figure 1 shows the geographic differences in the standardized HBsAg positive and HCV prevalence ratios in Japan. A base map of Japan indicating the name of prefectures is shown in the Supplementary Fig. 1. Municipalities with a high standardized HBsAg positive ratio tended to be seen often in Hokkaido and Okinawa. Many municipalities with a low standardized HCV prevalence ratio were seen, probably because the number of HCV carriers tended to be zero in many municipalities. Spearman’s correlation coefficient of the standardized HBsAg positive ratio and the standardized HCV prevalence ratio was 0.079 (*p*-value = 0.001), and there was a positive correlation between them.

Table 2 shows the basic characteristics of the data used in this study.

**Table 2:** Basic characteristics of the data used in this study

Characteristics	Median (Interquartile range)
Socioeconomic characteristics	
Proportion of fatherless households	1.3 (1.0—1.7)
Proportion of divorced persons	5.0 (4.3—5.8)
Proportion of persons with low educational level	23.2 (16.7—31.0)
Proportion of labourers	7.4 (6.6—8.4)
Proportion of unemployed persons	3.9 (3.2—4.6)
Taxable income per capita (Unit:1,000 yen)	1095.6 (922.9—1289.2)
Proportion of households living in rental housing	19.8 (12.9—27.2)
Other characteristics	
Population density	2.0 (0.6—7.4)
Proportion of non-Japanese persons	0.7 (0.4—1.3)
Proportion of elderly households	25.4 (21.2—31.2)
Proportion of single households	27.5 (22.8—32.8)
Proportion of self-employed persons	16.1 (11.3—22.9)
Number of hospitals per capita	5.9 (0.0—10.1)
Number of medical clinics per capita	68.4 (54.1—84.9)
HBV screening	
Number of participants	752 (280—2198)
Number of HBsAg-positive persons	6.0 (2.0—15.0)
HBsAg positive rate per 1,000 participants	6.2 (3.4—10.5)
HCV screening	
Number of participants	745 (278—2182)
Number of HCV carriers	2.0 (0.0—7.0)
HCV prevalence per 1,000 participants	2.3 (0.0—4.5)

*HBsAg* hepatitis B surface antigen, *HCV* hepatitis C virus

Table 3 shows the results of the principal component analysis, and the principal components 1–4 are shown. The eigenvalues of principal components 1 and 2 were relatively large. Principal component 1 can be interpreted as a component related to level of rurality because coefficients of variables related to urbanization tended to be below zero and large. In addition, principal component 2 can be interpreted as a component related to low socioeconomic status among individuals because only the coefficient of taxable income per capita is below zero. We used the first two principal components for deriving the socioeconomic deprivation level because the eigenvalues were higher than 1 for those principal components.

**Table 3:** Results of principal component analysis

Socioeconomic characteristics	Variable loadings			
	PC 1	PC 2	PC 3	PC 4
Proportion of fatherless households	− 0.570	0.657	− 0.084	0.214
Proportion of divorced persons	− 0.256	0.846	0.160	0.206
Proportion of persons with low educational level	0.780	0.397	0.097	0.212
Proportion of labourers	0.473	0.433	0.623	− 0.398
Proportion of unemployed persons	− 0.379	0.609	− 0.387	− 0.535
Taxable income per capita	− 0.609	− 0.615	0.307	− 0.145
Proportion of households living in rental housing	− 0.800	0.085	0.423	0.118
Eigenvalue	1.542	1.500	0.924	0.783
Proportion of variance explained	0.339	0.321	0.122	0.088
Cumulative proportion of variance explained	0.339	0.661	0.783	0.870

*PC* Principal component

Table 4 shows the posterior means of relative risk and the 95% CIs obtained by spatial Poisson regression analysis according to type of socioeconomic deprivation level. The deprivation level derived from the sum of the scores of principal components 1 and 2 was significantly and positively associated with HBsAg positivity and HCV prevalence. The deprivation level derived from the score of principal component 2 was also significantly and positively associated with the outcomes, whereas that derived from the score of principal component 1 was not associated with the outcomes. Population density was significantly and positively associated with HBsAg positivity and HCV prevalence when using the deprivation level derived from the score of principal component 1 and that derived from the sum of the scores of principal components 1 and 2.

**Table 4:** Results of posterior mean of relative risk by the spatial Poisson regression

	HBsAg positivity	HCV prevalence
Analysis method and explanatory variables	Relative risk (95% CI)	Relative risk (95% CI)
Analysis1 ^a^		
Population density	1.070 (1.014, 1.123)	1.128 (1.027, 1.242)
Proportion of non-Japanese persons	1.016 (0.978, 1.058)	1.020 (0.958, 1.101)
Proportion of elderly households	0.998 (0.937, 1.064)	0.910 (0.815, 1.010)
Proportion of single households	1.027 (0.977, 1.077)	1.011 (0.933, 1.098)
Proportion of self-employed persons	0.969 (0.921, 1.015)	1.017 (0.938, 1.102)
Number of hospitals per capita	0.990 (0.953, 1.032)	1.018 (0.949, 1.095)
Number of medical clinics per capita	1.002 (0.960, 1.042)	1.043 (0.976, 1.107)
Deprivation level	1.096 (1.030, 1.175)	1.211 (1.076, 1.342)
Analysis2 ^b^		
Population density	1.046 (0.994, 1.106)	1.041 (0.951, 1.167)
Proportion of non-Japanese persons	1.027 (0.988, 1.065)	1.043 (0.976, 1.120)
Proportion of elderly households	1.066 (1.005, 1.129)	1.071 (0.960, 1.184)
Proportion of single households	1.010 (0.959, 1.065)	0.972 (0.891, 1.068)
Proportion of self-employed persons	0.992 (0.940, 1.051)	1.085 (0.979, 1.205)
Number of hospitals per capita	0.988 (0.950, 1.027)	1.009 (0.935, 1.090)
Number of medical clinics per capita	0.971 (0.934, 1.011)	0.988 (0.922, 1.056)
Deprivation level	0.963 (0.898, 1.022)	0.880 (0.774, 1.007)
Analysis3 ^c^		
Population density	1.069 (1.006, 1.124)	1.108 (1.020, 1.198)
Proportion of non-Japanese persons	1.013 (0.977, 1.052)	1.017 (0.951, 1.094)
Proportion of elderly households	1.007 (0.959, 1.063)	0.937 (0.850, 1.026)
Proportion of single households	1.010 (0.961, 1.057)	0.991 (0.909, 1.082)
Proportion of self-employed persons	1.008 (0.960, 1.056)	1.099 (1.002, 1.203)
Number of hospitals per capita	0.986 (0.947, 1.025)	1.000 (0.934, 1.076)
Number of medical clinics per capita	1.002 (0.963, 1.044)	1.041 (0.977, 1.115)
Deprivation level	1.108 (1.058, 1.159)	1.254 (1.160, 1.373)

*CI* credible interval, *HBsAg* hepatitis B surface antigen, *HCV *hepatitis C virus, *PC* principal component

^a^Analysis using the deprivation level based on sum of scores of PC1 and PC2

^b^Analysis using the deprivation level based on score of PC1

^c^Analysis using the deprivation level based on score of PC2

## Discussion

This study revealed that risks for HBsAg positivity and HCV prevalence were associated with the municipal socioeconomic deprivation level among individuals who have never participated in hepatitis screening. There was an association between socioeconomic deprivation level and both viruses. Although it is known that the risk of liver cancer mortality differed depending on municipal socioeconomic deprivation level in Japan [16], this is the first time the association between the results of hepatitis virus screening and socioeconomic deprivation level has been shown in Japan. In addition, the socioeconomic deprivation level based on the principal component that is interpreted as low socioeconomic status among individuals was associated with HBsAg positivity and HCV prevalence. Thus, it is considered that the socioeconomic status of individuals living in a municipality, rather than the municipal level of rurality, is positively associated with the outcomes.

Although the geographical distribution of the outcomes differed, there was a positive correlation between them among municipalities. This positive correlation is considered to be related to the association of both the outcomes with the municipal socioeconomic deprivation level.

We briefly discuss possible reasons for the association shown in this study. Firstly, it is considered that HCV or HBV infection tends to occur among persons with low socioeconomic status or living in deprived areas. In Japan, blood transfusion was a major infection route for HBV and HCV in the twentieth century [30, 31], and continuous use of injection equipments is also known to be related to infection of HBV [32]. The sanitary environment of areas is related to the infection rate of these viruses, and infection by blood transfusion and injection equipments are still a major concern in developing countries [33]. Therefore, there is a possibility the infection rate from blood transfusion or injections may have varied depending on areal socioeconomic deprivation levels in Japan in the twentieth century, whereas an evidence supporting this hypothesis does not exist. Sexually transmitted infection is another HBV infection route in Japan [30]. There is a socioeconomic disparity in diagnosing sexually transmitted infections [34, 35], and a similar disparity might also exist for HBV in Japan. Moreover, awareness or perception of the HBV vaccine may differ depending on socioeconomic status. In China and Korea, socioeconomic inequality in hepatitis B vaccination has been shown [36–38], possibly due to income status and awareness of hepatitis B [36, 37]. However, infants of pregnant women whose HBsAg and Hepatitis B e antigen are positive began to be vaccinated by public expenses from 1986 in Japan [39]. Therefore, a disparity in vaccination rate depending on areal socioeconomic deprivation level might not be true for Japan.

An another explanation of the disparity, the treatment rate or awareness of infection for hepatitis viruses may differ depending on the socioeconomic deprivation level. In Japan, screening for hepatitis viruses is also conducted in workplaces [40], and implementation of the screening depends on the workplaces. Opportunities for participation in this screening may differ according to the type of occupation or workplace, and employees in large companies or of high socioeconomic status might have more opportunities to be screened. New methods of HCV treatment, such as direct-acting antivirals, have been developed in recent decades [41]. Therefore, if the hepatitis virus is detected early in workplaces, patients can receive treatment early. In addition, according to a study in Korea, self-awareness and family awareness of hepatitis virus infection differed depending on socioeconomic status [42]. Persons with higher socioeconomic status might know of the infection risk of themselves or their family members and have an early examination. An increase in treatment and awareness of infection in persons with high socioeconomic status may have reduced the prevalence of HCV and HBV in less deprived areas in Japan.

Population density was also positively associated with HBsAg positivity and HCV prevalence. Although the reason is uncertain, an association between urbanization level and the prevalence of viruses differs across countries. A study in India found that anti-HCV prevalence was associated with living in a rural area [43]. HBsAg prevalence was also higher in rural areas in a study conducted in Madagascar [44]. In contrast, a study in the Netherlands indicated that HCV prevalence was clustered in urban areas [11].

This study showed that the deprivation level increase relative risk of HBsAg positivity and HCV prevalence among individuals who have not participated in the hepatitis screening. Hepatitis virus screening on a larger scale in deprived areas will increase the detection of undiagnosed carriers, and early detection and treatment of virus carriers can reduce liver cancer and cirrhosis in Japan. Nevertheless, people in those deprived areas might be less likely to participate in testing for the hepatitis virus. In the United States, it was shown that people with less education, lower income, and private health insurance were significantly less likely to be screened for HCV [45]. It is meaningful to conduct an epidemiological study investigating whether the participation rate for hepatitis virus screening differs depending on socioeconomic deprivation levels. If the participation rate is low in highly deprived areas, an awareness program for informing the population of the merits of participation in screening is also needed. In addition, as discussed, the rate of HBV vaccination or awareness of the hepatitis virus may differ depending on the deprivation level. It will be useful to investigate those issues in the future.

There are some limitations for this study. First, we could not obtain data on gender in this study. The HBsAg positivity and HCV carrier rates are higher in middle-aged or older men than in women in Japan [5]. Geographic differences might differ by gender. Secondly, this was an ecological study. A study investigating the individual socioeconomic status and seropositive rates is needed to verify the results of this study. Thirdly, the study subjects had never participated in hepatitis virus screening. Those who have participated in screening before or who have been treated for the hepatitis virus are not included in the data. In addition, participation in hepatitis screening is not mandatory, and individuals voluntarily apply for the screening, and the attributes of participants and nonparticipants may be different. Moreover, number of target persons of the screening (sum of the participants and persons who have not participated in the screening) for each municipality is uncertain, and we could not calculate the participation rate in each municipality. A similar epidemiological study using randomly chosen individuals from each municipality will also be meaningful in the future. Fourthly, socioeconomic deprivation level of a municipality can change based on methods for deriving the level. Another method for deriving areal deprivation index using the Census data has been proposed in a previous study [46]. We confirmed that a similar socioeconomic disparity in the results of hepatitis screening was also observed when using the deprivation index proposed in the previous study [46]. Fifthly, participants aged 40 years old is quite high in the national screening because the main target of the screening is those aged 40 years old.

## Conclusion

We investigated the association between areal socioeconomic deprivation level and the HBsAg positivity and HCV prevalence using national screening data from Japanese municipalities. This ecological study, conducted using a spatial model, found that the municipal socioeconomic deprivation level was significantly and positively associated with HBsAg positivity and HCV prevalence among individuals who have never participated in hepatitis screening. Thus, participation in hepatitis virus screening is important and meaningful, particularly for areas with a high lower socioeconomic level in Japan.

## Supplementary Information

**Additional file 1:** **Supplementary Figure 1.** A base map of Japan with prefectural name

## Data Availability

The data used in this study can be obtained from websites of government statistics in Japan (The Report on Regional Public Health Services and Health Promotion Services. [cited24 January 2022]. Available from: https://www.e-stat.go.jp/stat-search/files?page=1&toukei=00450025&tstat=000001030884; State of prefectures and municipalities (System of social and demographic statistics). [cited 24 January 2022]. Available from: https://www.e-stat.go.jp/regional-statistics/ssdsview; Census data. [cited 24 January 2022]. Available from: https://www.e-stat.go.jp/stat-search/files?page=1&toukei=00200521; The digital national land information (Administrative area data). [cited 24 January 2022]. Available from: https://nlftp.mlit.go.jp/ksj/gml/datalist/KsjTmplt-N03-v3_0.html).

## Abbreviations

CL: Credible interval
CLEIA: Chemiluminescent enzyme immunoassay
HBsAg: Hepatitis B surface antigen
HBV: Hepatitis B virus
HCV: Hepatitis C virus
PC: Principal component
