# Supplementary figures and images for: Analysis of the association between areal socioeconomic deprivation levels and viral hepatitis B and C infections in Japanese municipalities
Source: BMC Public Health. 2022 Apr 7;22:681. doi: 10.1186/s12889-022-13089-w (PMC8991792; doi:10.1186/s12889-022-13089-w)

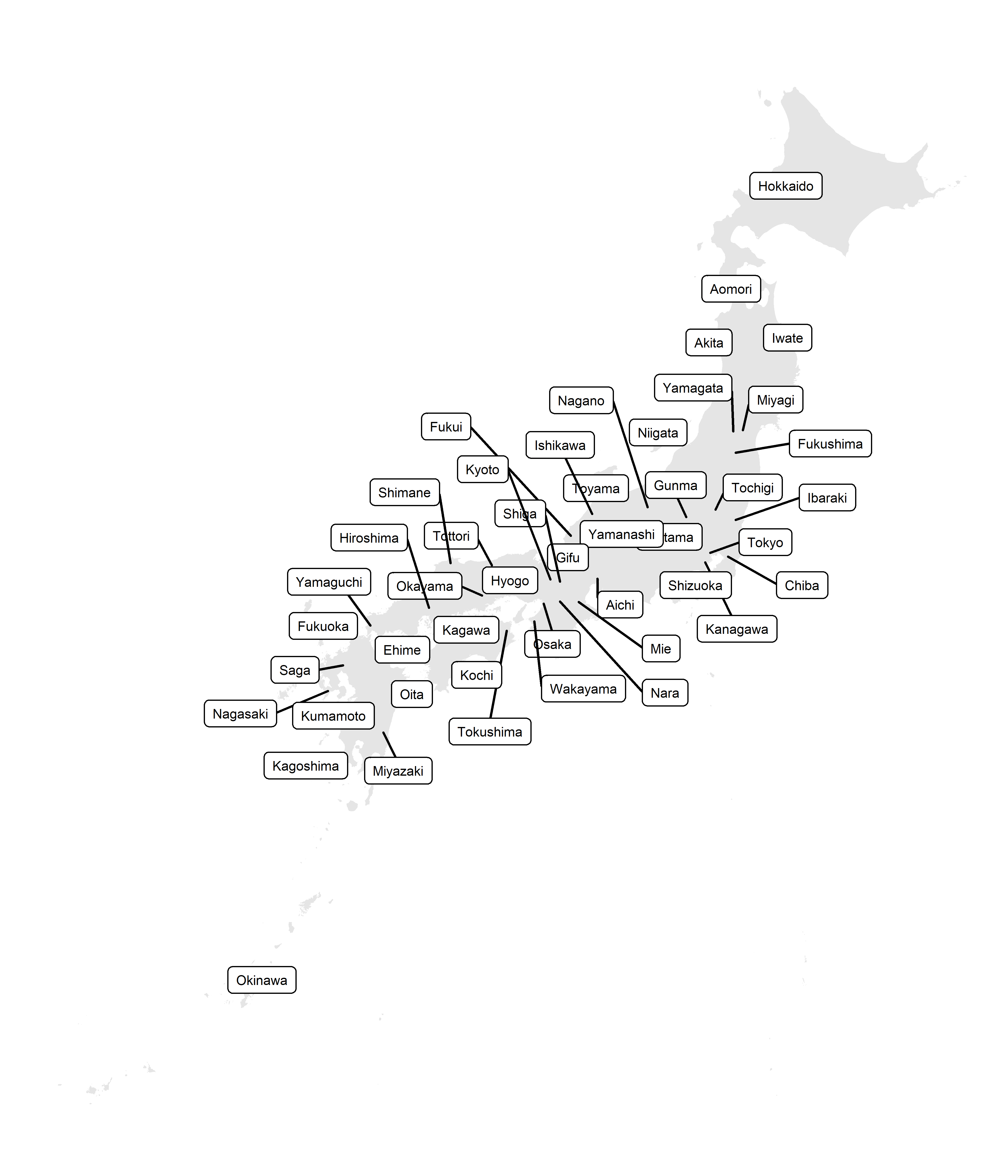

Supplement: Supplementary file 1 — Additional file 1: Supplementary Figure 1. A base map of Japan with prefectural name [file 12889_2022_13089_MOESM1_ESM.docx]
